# Malignant Potential of Endobronchial Glomangioma: A Case Report of an Unpredictable Diagnosis and Unexpected Outcome

**DOI:** 10.2174/0118743064397717250715093022

**Published:** 2025-07-23

**Authors:** Ginanjar Arum Desianti, I. Wayan Pande Adhyaksa, Eylin Halim Rahardjo, Agung Wibawanto

**Affiliations:** 1 Faculty of Medicine, Department of Pulmonology and Respiratory Medicine, Universitas Indonesia - Persahabatan National Respiratory Referral Hospital, Jakarta, Indonesia; 2 Faculty of Medicine, Department of Pulmonology and Respiratory Medicine, Universitas Indonesia, Jakarta, Indonesia; 3 Department of Cardiovascular and Thoracic Surgery, Persahabatan National Respiratory Referral Hospital, Jakarta, Indonesia

**Keywords:** Endobronchial, Lung, Glomus tumor, Glomangioma, Malignant, Outcome

## Abstract

**Background:**

Glomus tumors are neoplasms typically arising from the glomus body in the skin or subcutaneous tissue. They are rarely found in visceral organs, including the respiratory tract. Glomangioma is a vascular variant, and its pulmonary subtype is challenging to predict due to the absence of specific symptoms or distinctive radiological features. While most glomangiomas are benign, in rare cases, they can exhibit aggressive clinical and histological characteristics, leading to severe conditions.

**Case presentation:**

We report a case of malignant endobronchial glomangioma in a patient presenting with hemoptysis and atypical chest pain. Chest computed tomography (CT) revealed an endobronchial tumor in the left distal main stem bronchus, partially obstructing the lumen. The patient was clinically diagnosed with suspected metastatic lung cancer and was scheduled for bronchoscopy and biopsy. During the biopsy, a rounded, bulging mass was partially removed; however, significant intraoperative bleeding occurred, necessitating the formation of an intentional blood clot. Ongoing bleeding and airway necessitated emergency pneumonectomy after 24 hours. Unfortunately, the patient experienced cardiac arrest postoperatively and died. Pathological examination revealed a mass with prominent vascular components lined by endothelial cells, with immunohistochemistry showing positivity only for smooth muscle actin, supporting the diagnosis of glomangioma.

**Conclusion:**

Although difficult to diagnose clinically prior to biopsy, malignant endobronchial glomangioma should be considered in certain patient populations due to its specific outcomes and complications. Preventive measures and targeted interventions should be implemented to manage iatrogenic bleeding complications associated with biopsy procedures.

## 1 INTRODUCTION

Glomus tumor is a predominantly benign neoplasm originating from the glomus body, which is part of the thermoregulation pathway in the dermis [1]. It is rarely found as an extracutaneous tumor and is commonly found in the upper extremities [2]. There are several case reports that have documented extracutaneous glomus tumors in the trachea and lungs, but the exact incidence remains unknown [3-7]. In this report, we describe a case of uncertain malignant potential of endobronchial glomangioma, presenting with hemoptysis. The diagnosis of glomangioma was unexpected, and the patient experienced severe complications and a poor outcome regarding the treatment process.

## 2 CASE REPORT

A 45-year-old male presented to the clinic with a chief complaint of occasional hemoptysis one month before admission. He also experienced chest pain and tightness, particularly in the left chest and shoulder, which worsened with physical activity. He had no other symptoms, such as shortness of breath, fever, weight loss, or hoarseness. His medical history included tuberculosis eight years ago, and he was declared cured. He had no history of hypertension, diabetes mellitus, hematological disease, and did not consume any antiplatelet or anticoagulant drugs. There was no family history of malignancy. Upon examination, his vital signs were stable, but breath sounds were diminished in the left lower lung field. There were no other physical abnormalities. He had previously undergone thoracentesis at another hospital, which yielded less than 100 cc of exudative pleural fluid. However, no cytological or microbiological data on the pleural fluid examination were available.

**Fig. (1):**
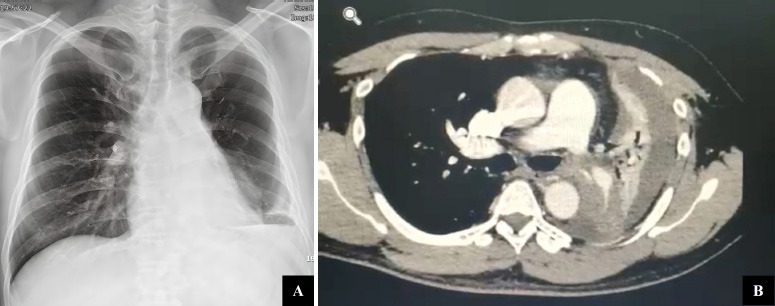
**(A)** Blunting of the left costophrenic angle on chest X-ray. **(B)** Chest CT showed an endobronchial tumor in the left main stem bronchus, with lower lobe atelectasis and mild to moderate pleural effusion.

His chest X-ray showed mediastinal widening with blunting of the left costophrenic angle (Fig. **1A**). Chest computed tomography (CT) revealed an endobronchial tumor in the left distal main stem bronchus that partially blocked the lumen and caused lingular and lower lobe atelectasis with mild to moderate pleural effusion (Fig. **1B**). The CT scan also showed multiple mediastinal and hilar lymphadenopathies at the level of 2R-L, 4R-L, 5, 6, and 9. The patient was clinically diagnosed with suspected metastatic lung cancer. He was planned to undergo bronchoscopy and biopsy as a diagnostic procedure. The hematology profile before the procedure was within normal limits.

**Fig. (2):**
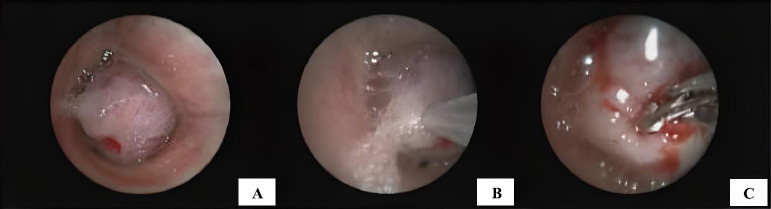
Bronchoscopic view of the endobronchial mass: **(A)** Endobronchial mass obstructing the lumen of the left main stem bronchus; **(B)** Initial tissue sampling using a needle aspiration technique; **(C)** Rigid forceps biopsy performed on the mass surface.

Bronchoscopy was performed under general anesthesia using fentanyl and propofol. We identified a round, bluish, and hypervascular mass that almost completely blocked the left main stem bronchus, located approximately 4 cm from the carina (Fig. **2A**). Due to the high risk of bleeding, needle aspiration and forceps biopsy were initially performed (Fig. **2B**, **2C**). After three biopsy attempts for each procedure, no prominent bleeding occurred, but rapid on-site evaluation (ROSE) did not provide a conclusive result. To obtain a larger sample, we planned to perform a biopsy using a 1.7 mm cryoprobe with a 5-second freezing time. The rounded and bulging mass was successfully removed, but there was significant intraoperative bleeding.

**Fig. (3):**
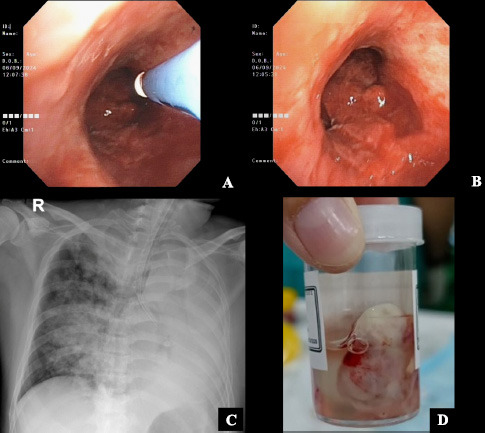
Intraoperative and postoperative findings following bronchoscopic intervention: **(A)** Argon plasma coagulation (APC) applied to control tumor bleeding; **(B)** Intentional endobronchial blood clot for tamponade; **(C)** Post-procedure chest X-ray showing complete left lung atelectasis, right lower lobe opacities, and double-lumen endotracheal tube placement; **(D)** Gross appearance of the tumor tissue obtained using a cryoprobe biopsy.

Immediate hemostatic procedures were undertaken, including instillation of diluted adrenaline solution and cold saline, as well as the wedging method using a rigid scope and argon plasma coagulation (Fig. **3A**). We also ensured clearance of the right lung bronchus and prevented blood flooding. Despite these interventions, the bleeding continued profusely, with a total blood loss of 200 cc. To control the bleeding, we decided to create an intentional blood clot as a barrier at the proximal site of the bleeding source (Fig. **3B**). We also used a double-lumen endotracheal tube with a bronchial blocker to secure the right airway and administered a combination of hemostatic drugs, including carbazochrome and tranexamic acid. A post-procedure chest X-ray showed total left lung atelectasis with airspace opacities in the right lower lobe (Fig. **3C**).

**Fig. (4):**
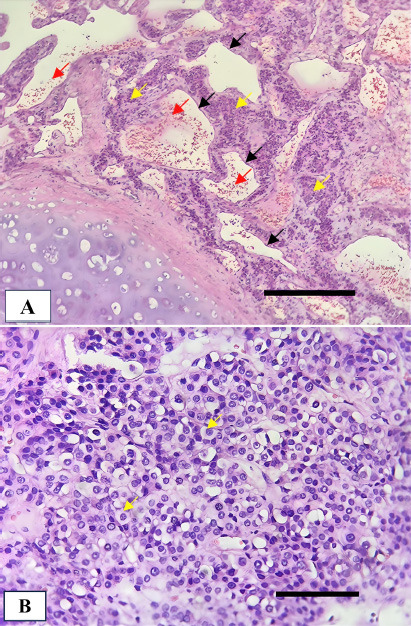
Histopathological features of the endobronchial tumor stained with hematoxylin and eosin (H&E): **(A)** Prominent vascular components lined by endothelial cells (black arrows) and filled with erythrocytes (red arrows). Tumor cells are distributed between the vascular branches (yellow arrows) (100x magnification; scale bar: 400 µm); **(B)** Clusters of uniform round tumor cells with scant cytoplasm (400x magnification; scale bar: 100 µm).

Macroscopically, the tumor measured >2.0 cm and was located in the deep subepithelial tissue (Fig. **3D**). Pathological examination showed a well-defined tumor mass consisting of proliferative blood vessels in various shapes and sizes, lined with a layer of endothelial cells. The vascular lumen was filled with erythrocytes (Fig. **4A**). The tumor tissue was surrounded by respiratory epithelium, with cell proliferation observed between the vessels and within the stroma. The cells had round to oval-shaped nuclei, no nuclear atypia, were relatively monotonous, had smooth chromatin, and displayed partially clear eosinophilic cytoplasm. Further, atypical mitotic activity was difficult to identify (Fig. **4B**). The stroma was generally hyalinized and partly myxoid. These characteristics were consistent with a glomus tumor (glomangioma type), with low-grade typical carcinoid tumor as a differential diagnosis.

**Fig. (5):**
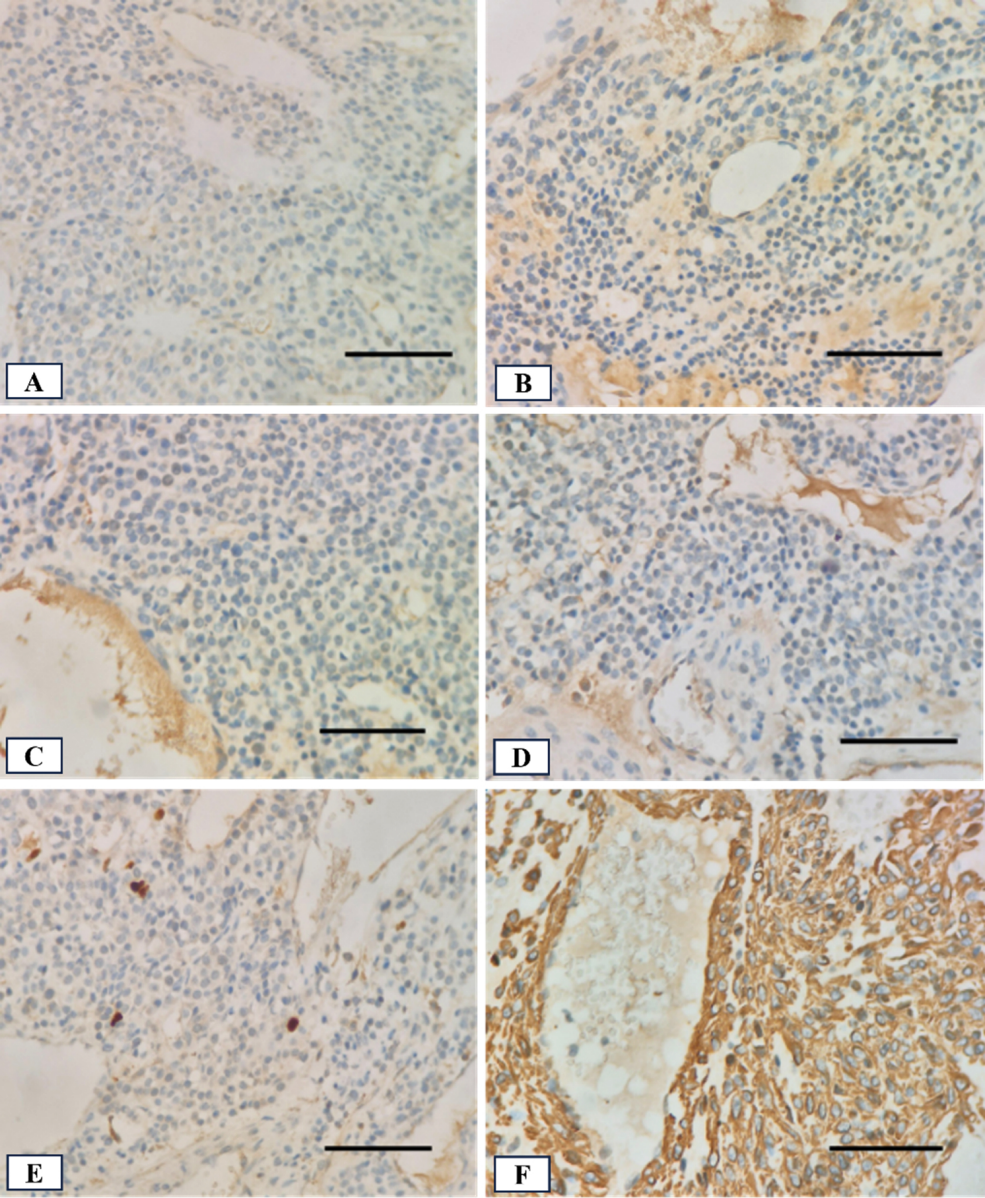
Immunohistochemical staining of the tumor tissue with hematoxylin counterstain (400x magnification; scale bar: 100 µm in all panels): **(A)** TTF-1 shows negative staining; **(B)** Syn and **(C)** CgA both show negative staining; **(D)** AE1-3 shows negative staining; **(E)** Ki-67 staining shows a low proliferative index; **(F)** SMA staining is strongly positive.

Immunohistochemical results showed a negative result for thyroid transcription factor 1 (TTF-1), synaptophysin (Syn), and chromogranin (CgA) (Fig. **5A-C**). Those are markers for primary lung adenocarcinoma and neuroendocrine cells [8, 9]. The tumor also tested negative for cytokeratin AE1/AE3 (AE1-3) and Ki67, thus ruling out an epithelial origin and indicating a low index of proliferation (Fig. **5D**, **5E**). The only positive marker was smooth muscle actin (SMA) (Fig. **5F**). It was concluded that the tumor was a glomangioma. Although the tumor size exceeded 2.0 cm and was in a deep site location, the absence of nuclear atypia and mitotic activity led to its classification as an uncertain malignant potential.

**Fig. (6):**
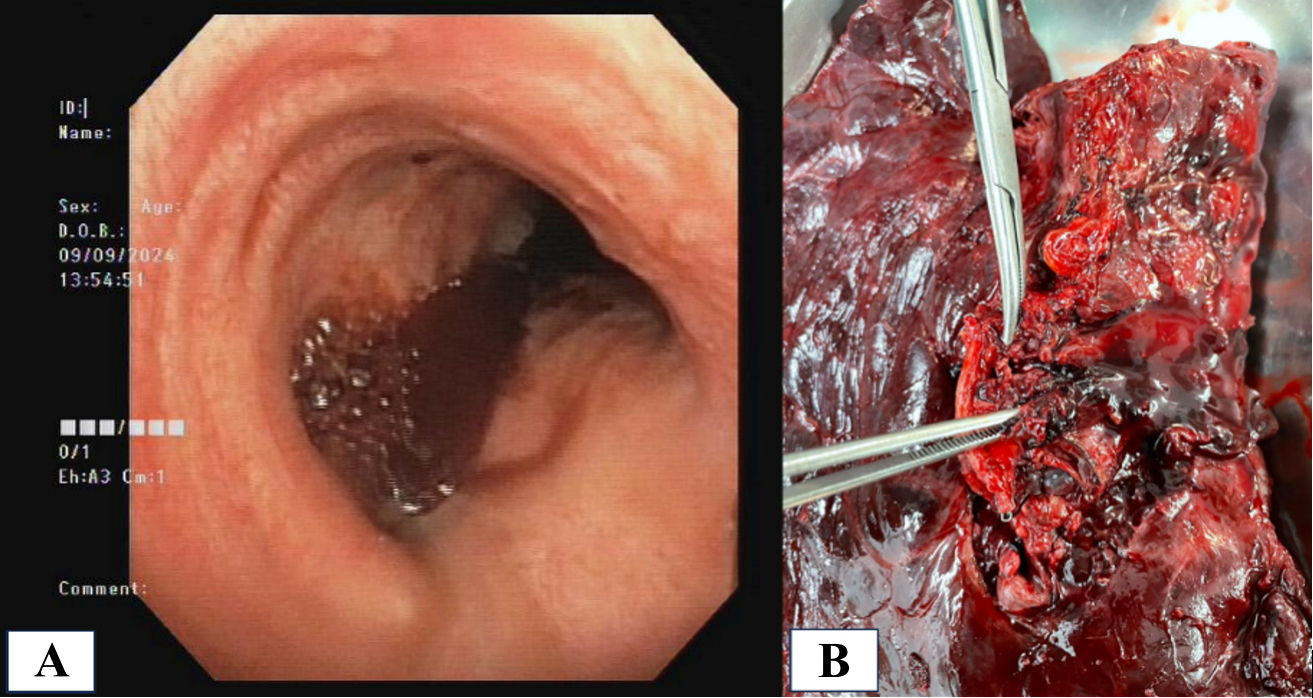
Intraoperative findings: **(A)** Bronchoscopic view showing a large blood clot in the left main stem bronchus, with ongoing blood flow visible to the right lung; **(B)** Resected left lung specimen following thoracotomy pneumonectomy.

After observation for 24 hours in the intensive care unit, we planned to perform a bronchoscopy to reevaluate the bleeding. A large blood clot was found completely obstructing the left main bronchus lumen, with residual blood clot spread throughout the right main bronchus branches (Fig. **6A**). This finding suggested ongoing bleeding, so we decided to perform a lung resection (Fig. **6B**). Due to extensive damage, the patient underwent a left thoracotomy pneumonectomy surgery. Mediastinal lymphadenopathies were also removed. Intraoperatively, the bronchoscopy, surgery, and anesthesia teams struggled to maintain the patient’s hemodynamic stability, since the bleeding still remained and blocked airway ventilation. The procedure was completed, and the patient was admitted to the intensive care unit postoperatively. Unfortunately, 8 hours after surgery, the patient experienced cardiac arrest and died.

## 3 DISCUSSION

Glomus tumors are rare neoplasms arising from the glomus body, a structure involved in the thermoregulatory pathway, composed of glomus cells, vasculature, and smooth muscle cells [1, 2]. Histologically, they are classified into three main variants: solid glomus tumors (75%), glomangiomas (20%), and glomangiomyomas (5%) [1, 10]. Solid types have minimal vascular and smooth muscle components, glomangiomas show prominent vasculature, and glomangiomyomas feature both vasculature and spindle-shaped smooth muscle cells [1, 3]. Malignant variants are extremely rare [1].

While typically found in the digits, glomus tumors can also occur in visceral organs, including the respiratory tract [2, 10]. Primary pulmonary glomus tumors, particularly in the trachea and lung, are uncommon and usually present in individuals aged 20–40 years, with solid tumors comprising the majority (81.8%) [9]. Pulmonary glomangiomas tend to present later, around age 43–45, and show a marked male predominance (7:1), consistent with our case [11].

Our patient presented with chest pain, a common symptom of pulmonary glomus tumors, along with other possible signs, such as cough, hemoptysis, and dyspnea [6, 12-14]. Imaging showed left lung consolidation without specific features. Due to their nonspecific radiologic appearance, modalities like chest X-ray, CT, and FDG-PET have limited diagnostic value [4, 15]. Some cases may benefit from contrast enhancement due to tumor vascularity [11]. Pulmonary glomus tumors often mimic both benign and malignant endobronchial masses, commonly suspected as carcinoid tumors [6, 16]. Therefore, diagnosis is typically definitive and requires histopathologic confirmation via bronchoscopy [4, 6].

Intrabronchial characteristics vary by tumor extension, with most presenting as hypervascular masses obstructing the airway [17, 18]. In our case, significant hemorrhage was anticipated, prompting an initial biopsy using an aspiration needle and forceps to minimize bleeding risk. However, the pathological results were inconclusive, which led us to proceed with a cryoprobe biopsy using a 5-second freeze. This resulted in mass detachment and severe bleeding. While a 3–4 second freeze is typically sufficient, longer durations are associated with larger tissue samples [19, 20]. Although Zhang *et al.* [12] recommend avoiding biopsy in hypervascular tumors, definitive diagnosis remains crucial. To control the iatrogenic bleeding, we employed vasoconstrictors (cold saline, diluted adrenaline), coagulation agents, the wedging method, and argon plasma coagulation; however, hemostasis remained difficult. Eventually, clot formation served as a barrier, preventing blood from entering the contralateral lung [21]. A double-lumen endobronchial tube with a bronchial blocker was then used to isolate and protect the unaffected lung [22].

There are three categories of glomus tumors based on the World Health Organization (WHO) classification: benign, uncertain malignant potential, and malignant [23, 24]. This classification is determined by several criteria, including tumor location, size, nuclear atypia, mitotic activity, and the presence of atypical mitotic figures Table **1** [6, 25]. Most glomus tumors are benign and occur in superficial sites [13]. In our case, the tumor met WHO criteria for uncertain malignant potential due to its size exceeding 2.0 cm and deep endobronchial location, despite lacking atypia or high mitotic activity. Histologically, the tumor was identified as a glomangioma subtype, though morphologic overlap with other pulmonary neoplasms, such as carcinoid or neuroendocrine tumors, often requires immunohistochemical (IHC) confirmation. In this patient, IHC staining showed strong positivity for smooth muscle actin (SMA) and negativity for cytokeratin AE1/AE3, Ki67, TTF-1, synaptophysin, and chromogranin A, excluding both epithelial and neuroendocrine origins [26-28]. Notably, SMA and vimentin are recognized as hallmark markers for glomangioma diagnosis, as supported by previous reports [13, 29].

Surgical resection remains the first-line treatment for glomus tumors, especially in cases of life-threatening airway obstruction or uncontrolled hemorrhage. However, rigid bronchoscopy with debulking techniques—such as electrocautery, laser ablation, or cryotherapy—has been reported as effective in selected patients [12, 30, 31]. In patients with strong clinical suspicion, a conservative approach may help avoid surgical risks and financial burden. Nevertheless, it carries the risks of tumor rupture, progressive obstruction, or malignant transformation [12]. In our case, bronchoscopy identified and partially removed a bulging endobronchial mass. However, the procedure was complicated by severe bleeding due to the tumor’s rich vascular supply. Despite multiple hemostatic efforts, the bleeding persisted. Consequently, a left thoracotomy pneumonectomy was performed after 24 hours of observation. This contrasts with the findings of Ho *et al.*, who reported successful management of glomus tumors with bronchoscopy alone [32]. Our case highlights the limitations of endoscopic treatment in tumors that are deeply located, highly vascular, and potentially malignant. Early recognition of bleeding risk is therefore critical for guiding appropriate management.

Several studies have proposed adjunctive strategies to minimize procedural risks in hypervascular endobronchial tumors. Tanaka *et al.* demonstrated that transbronchoscopic diode laser ablation provides effective photocoagulation for bleeding-prone airway lesions, offering a minimally invasive hemostatic option [33]. Similarly, Zhou *et al.* reported that preoperative embolization in patients undergoing lung surgery for destruction significantly reduces intraoperative blood loss and operative complexity [34]. Neither modality was employed in our case, which may have contributed to the difficulty in achieving bleeding control. This highlights the importance of thorough pre-procedural planning, incorporating advanced bronchoscopic technologies and vascular management strategies to improve safety and optimize outcomes in the treatment of complex endobronchial glomus tumors. To contextualize our findings, we reviewed and summarized previous case reports of endobronchial and tracheal glomangioma as presented in Table **2**.

## 4 STUDY LIMITATION

Nonetheless, this case report has limitations. As a single case, it cannot be generalized or used to determine optimal management strategies for endobronchial glomangioma. The lack of preoperative embolization and advanced endobronchial tools, such as the diode laser, due to resource constraints, limits assessment of their potential benefits. Retrospective interpretation may also introduce observer bias.

## CONCLUSION

Endobronchial glomangiomas present significant diagnostic and therapeutic challenges, particularly when located deep within the airway and exhibiting marked vascularity. Clinicians should maintain a high index of suspicion in patients with atypical endobronchial masses and hemoptysis, particularly when imaging is inconclusive. Although biopsy carries a significant bleeding risk, histopathologic confirmation is crucial. Management must be individualized and based on careful pre-procedural planning. While rigid bronchoscopy may be appropriate in selected cases, surgery remains the definitive treatment, particularly in life-threatening situations.

## Figures and Tables

**Table 1:** WHO classification of glomus tumor [23].

**Malignant**	**Uncertain Malignant Potential**	**Benign**
Nuclear atypia and any level of mitotic activity (>5 mitoses/50 high power field) or atypical mitotic figures	Not fulfilling malignant criteria. One or more atypical features other than nuclear polymorphisms, *e.g.*, size >2.0 cm and deep site location (absence of nuclear atypia)	None of the criteria for malignancy and/or malignant potential

**Table 2:** Summary of published cases of endobronchial and tracheal glomangioma.

**Author, Year, Refs.**	**Age/Sex**	**Symptoms**	**Tumor Location**	**Size (cm)**	**Diagnostic Method**	**Treatment**	**Follow-up**
Haraguchi *et al.*, 1991 [35]	61/M	Asymptomatic	Mid-trachea	1.2	Bronchoscopy, biopsy	Tracheal resection	No recurrence
Shin *et al.*, 1990 [36]	47/F	Cough, hemoptysis	Lower trachea	1.5×1.0×1.0	CT, bronchoscopy	Tracheal resection	No recurrence (13 mo)
Garcia-Prats *et al.*, 1991 [37]	58/M	Dyspnea, cough, hemoptysis	Mid-trachea	2.5	CT, bronchoscopy	Tracheal resection	No recurrence
Arapantoni *et al.*, 1995 [38]	65/M	Dyspnea, hemoptysis	Lower trachea	4.5×3	CT, bronchoscopy	Tracheal resection	No recurrence
Koskinen *et al.*, 1998 [39]	66/M	Asymptomatic	Lower trachea	3×2	Bronchoscopy	Endoscopic resection + Nd-YAG	Not stated
Watanabe *et al.*, 1998 [40]	43/M	Hoarseness	Lower trachea	2.0×1.6	CT, bronchoscopy	Tracheal resection	No recurrence (20 mo)
Menaissy *et al.*, 2000 [41]	34/M	Hemoptysis	Mid-trachea	2.4×2.1×1.6	CT, bronchoscopy	Tracheal resection	No recurrence (4 mo)
Gowan *et al.*, 2001 [42]	73/M	Cough, dyspnea, chest pain	Mid-trachea	1.6×0.6×0.3	CT, bronchoscopy	Tracheal resection	No recurrence (6 yrs)
Chien *et al.*, 2003 [43]	50/F	Cough, dyspnea, hemoptysis	Lower trachea	2.5×2.5×2	CT, bronchoscopy	Tracheal resection	No recurrence (1 yr)
Nadrous *et al.*, 2004 [44]	39/M	Intermittent hemoptysis	Upper trachea	2.0×1.5×1.5	CT, bronchoscopy	Tracheal resection	No recurrence (3 mo)
Haver *et al.*, 2008 [45]	10/F	Dyspnea	Mid-trachea	1.8×1.3×1.3	CT, bronchoscopy	Tracheal resection	No recurrence (2 yrs)
Colaut *et al.*, 2008 [46]	70/M	Dyspnea	Mid-trachea	2×1×1	CT, bronchoscopy	Endoscopic resection + Nd-YAG	No recurrence (2 yrs)
Parker *et al.*, 2010 [47]	43/F	Chest pain, asthma	Lower trachea	2.0×1.6×1.5	CT, bronchoscopy	Tracheal resection	Not stated
Shang *et al.*, 2010 [48]	59/M	Chest pain, dyspnea	Lower trachea	2.0×1×0.5	CT, bronchoscopy	Endoscopic resection	No recurrence (12 mo)
Fukumitsu *et al.*, 2023 [49]	34/F (pregnant)	Wheezing, dyspnea	Lower trachea	~2.1 cm	CT, bronchoscopy	Endoscopic resection	Under follow-up
Oide *et al.*, 2016 [15]	52/M	Cough, hemoptysis	Right mainstem bronchus	1.4×1.6×2.2	CT, bronchoscopy, biopsy	Surgical resection	No recurrence
**Present Case**	58/M	Hemoptysis, dyspnea	Left mainstem bronchus	>2 cm	CT, bronchoscopy, biopsy (cryoprobe)	Emergency pneumonectomy	Died post-op

Footnotes:

CT: Computed Tomography

Nd-YAG: Neodymium-doped Yttrium Aluminum Garnet laser

mo: months

yrs: years

Follow-up: Duration and outcome following treatment

## Data Availability

All data supporting this study are included within the article.

## LIST OF ABBREVIATIONS

AE1-3: = AE1/AE3
CgA: = Chromogranin A
CT: = Computed tomography
FDG-PET: = Fluorodeoxyglucose positron emission tomography
H&E: = Hematoxylin and eosin
MSA: = Muscle-specific actin
ROSE: = Rapid on-site evaluation
SMA: = Smooth muscle actin
Syn: = Synaptophysin
TTF-1: = Thyroid transcription factor 1
WHO: = World Health Organization

## STANDARDS OF REPORTING

CARE guidelines have been followed.
